# Complete genome sequence of *Mesorhizobium ciceri bv. biserrulae* type strain (WSM1271^T^)

**DOI:** 10.4056/sigs.4458283

**Published:** 2013-12-31

**Authors:** Kemanthi Nandasena, Ron Yates, Ravi Tiwari, Graham O’Hara, John Howieson, Mohamed Ninawi, Olga Chertkov, Chris Detter, Roxanne Tapia, Shunseng Han, Tanja Woyke, Sam Pitluck, Matt Nolan, Miriam Land, Konstantinos Liolios, Amrita Pati, Alex Copeland, Nikos Kyrpides, Natalia Ivanova, Lynne Goodwin, Uma Meenakshi, Wayne Reeve

**Affiliations:** 1 Centre for Rhizobium Studies, Murdoch University, Western Australia, Australia; 2 Los Alamos National Laboratory, Bioscience Division, Los Alamos, New Mexico, USA; 3 DOE Joint Genome Institute, Walnut Creek, California, USA; 4 Oak Ridge National Laboratory, Oak Ridge, Tennessee, USA; 5 Department of Agriculture and Food, Western Australia, Australia

**Keywords:** root-nodule bacteria, nitrogen fixation, evolution, lateral gene transfer, integrative and conjugative elements, symbiosis, *Alphaproteobacteria*

## Abstract

*Mesorhizobium ciceri* bv. *biserrulae* strain WSM1271^T^ was isolated from root nodules of the pasture legume *Biserrula pelecinus* growing in the Mediterranean basin. Previous studies have shown this aerobic, motile, Gram negative, non-spore-forming rod preferably nodulates *B*. *pelecinus* – a legume with many beneficial agronomic attributes for sustainable agriculture in Australia. We describe the genome of *Mesorhizobium ciceri* bv. *biserrulae* strain WSM1271^T^ consisting of a 6,264,489 bp chromosome and a 425,539 bp plasmid that together encode 6,470 protein-coding genes and 61 RNA-only encoding genes.

## Introduction

The productivity of sustainable agriculture around the world is heavily dependent on the provision of bioavailable nitrogen (N) [1]. The demand for N by non-leguminous and leguminous plants can be supplied by the application of chemically synthesized nitrogenous fertilizer onto crops and pastures. However, the production of fertilizer is costly and requires the burning of fossil fuels in the manufacturing process which increases greenhouse gas emissions. Furthermore, high application rates of fertilizer can contaminate ecosystems and waterways, and result in leaching into the environment.

In contrast, the demand for N by leguminous plants can be sustainably met through the biological process of N fixation that occurs following the successful formation of an effective symbiosis. This symbiotic nitrogen fixation (SNF) process can account for approximately 70% of the bioavailable nitrogen supplied to legumes [1].

One legume that has many beneficial agronomic attributes is *Biserrula pelecinus* L*.,* which is an annual herbaceous legume native to the Mediterranean basin that was introduced into Australian soil in 1994 [2]. The beneficial agronomic attributes of this legume include drought tolerance, hard seed production, easy harvesting characteristics, insect tolerance and most importantly, a capacity to grow well in the acidic duplex soils of Australia [2,3]. This monospecific legume specifically forms an effective nitrogen fixing symbiosis with the root nodule bacterium *Mesorhizobium ciceri* bv. *biserrulae* type strain WSM1271^T^ (= LMG23838 = HAMBI2942) [4,5]. Australian indigenous rhizobial populations were found to be incapable of nodulating *B. pelecinus* L [2]. However, within six years of the introduction of the inoculant into Australia, the *in situ* evolution of a diverse range of competitive strains capable of nodulating *B. pelecinus* L. compromised optimal N_2_-fixation with this host. This rapid emergence of less effective strains threatens the establishment of this legume species in the Australian agricultural setting. The sub-optimal strains appear to have evolved from indigenous mesorhizobia that acquired the island of genes associated with symbiosis from the original inoculant, WSM1271^T^, following a horizontal gene transfer event [6].

In this report, a summary classification and a set of general features for *M. ciceri* bv. *biserrulae* strain WSM1271^T^ are presented together with the description of the complete genome sequence and its annotation.

## Classification and features

**Figure 1:**
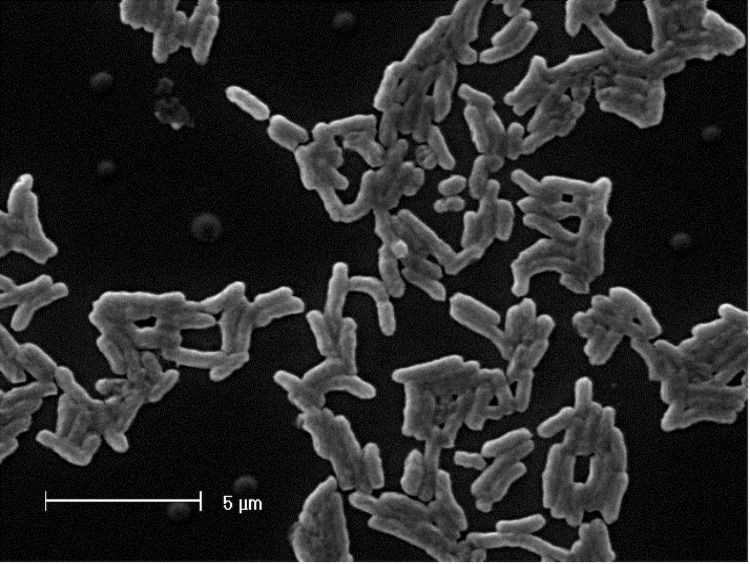
Image of *Mesorhizobium ciceri* bv. *biserrulae* strain WSM1271^T^ using scanning electron microscopy.

**Figure 2:**
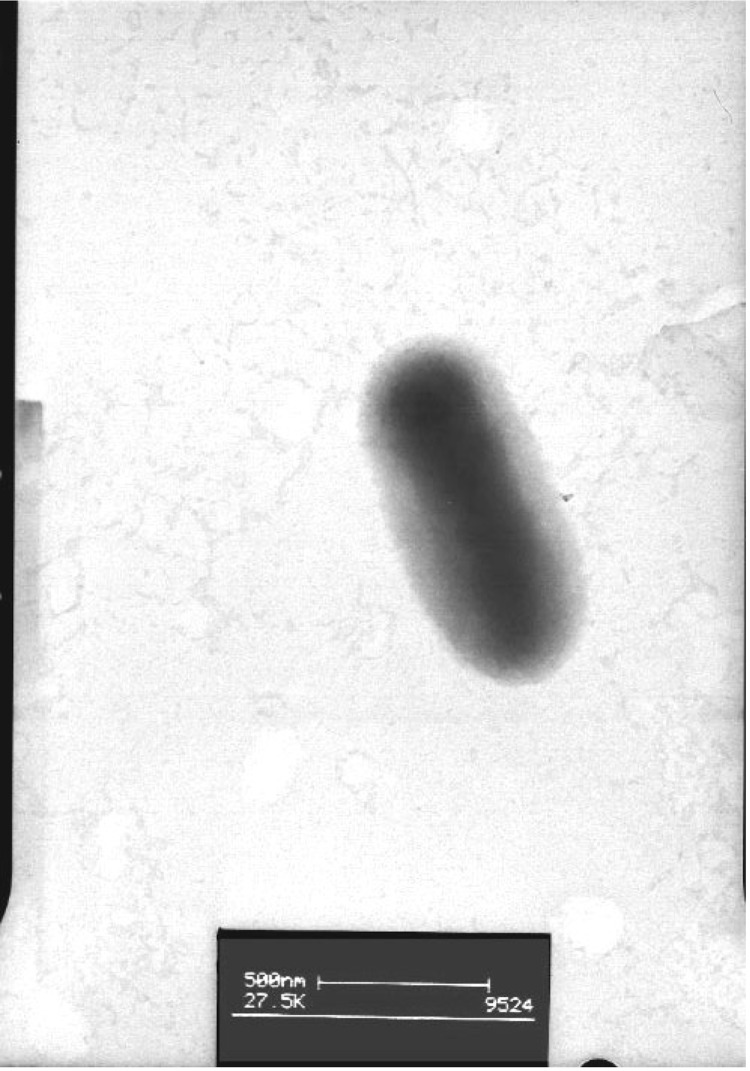
Image of *Mesorhizobium ciceri* bv. *biserrulae* strain WSM1271^T^ using transmission electron microscopy.

**Figure 3:**
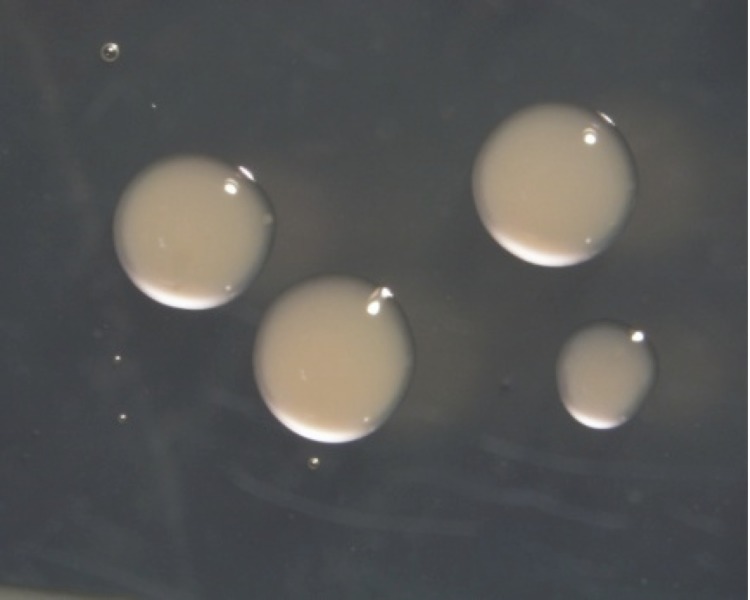
Image of *Mesorhizobium ciceri* bv. *biserrulae* strain WSM1271^T^ using the appearance of colony morphology on solid media.

*M. ciceri* strain WSM1271^T^ is a motile, Gram-negative, non-spore-forming rod (Figure 1 and Figure 2) in the order *Rhizobiales* of the class *Alphaproteobacteria*. They are moderately fast growing, forming 2-4 mm diameter colonies within 3-4 days, and have a mean generation time of 4-6 h when grown in half Lupin Agar (½LA) broth [7] at 28 °C. Colonies on ½LA are white-opaque, slightly domed, moderately mucoid with smooth margins (Figure 3).

**Figure 4:**
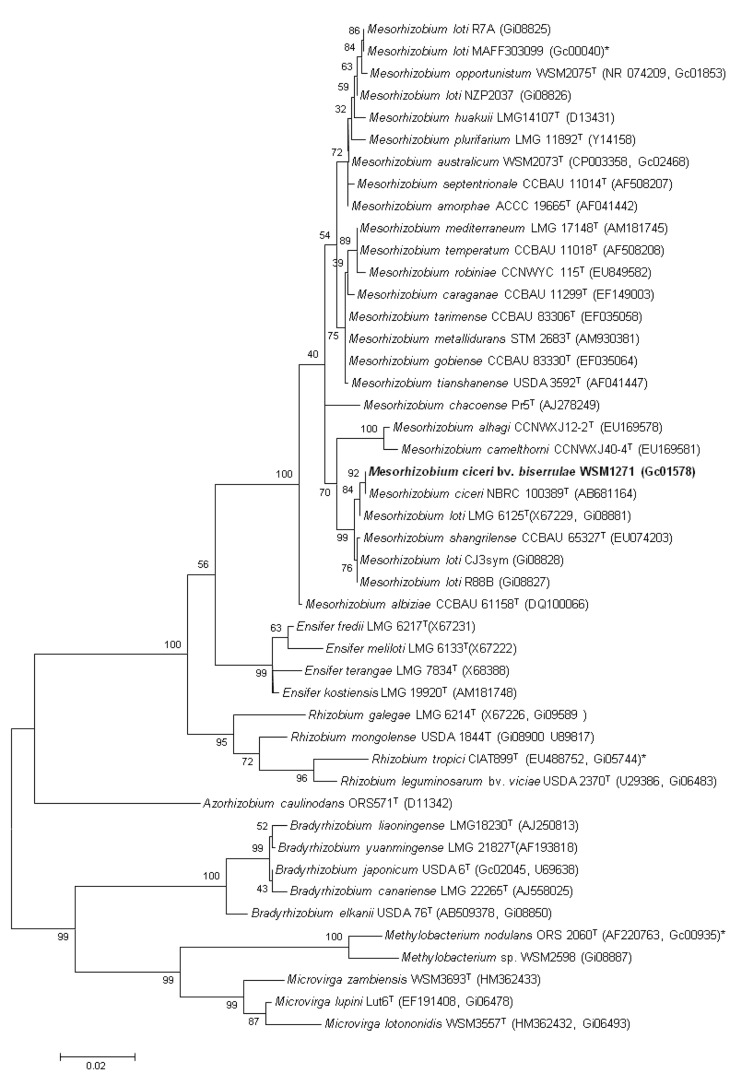
Phylogenetic tree showing the relationships of *Mesorhizobium ciceri* bv. *biserrulae* WSM1271^T^ (shown in bold print) with root nodule bacteria in the order *Rhizobiales* based on aligned sequences of the 16S rRNA gene (1,290 bp internal region). All sites were informative and there were no gap-containing sites. Phylogenetic analyses were performed using MEGA [19]. The tree was built using the Maximum-Likelihood method with the General Time Reversible model. Bootstrap analysis [20] was performed with 500 replicates to assess the support of the clusters. Type strains are indicated with a superscript T. Brackets after the strain name contain a DNA database accession number and/or a GOLD ID (beginning with the prefix G) for a sequencing project registered in GOLD [21]. Published genomes are indicated with an asterisk.

The organism tolerates a pH range between 5.5 and 9.0. Carbon source utilization and fatty acid profiles have been described before [6]. Minimum Information about the Genome Sequence (MIGS) is provided in Table 1. Figure 4 shows the phylogenetic neighborhood of *M. ciceri* bv. *biserrulae* strain WSM1271^T^ in a 16S rRNA sequence based tree. This strain clustered in a tight group, which included *M. australicum*, *M. ciceri*, *M. loti* and *M. shangrilense* and had >99% sequence identity with all four type strains. Our polyphasic taxonomic study indicates that WSM1271^T^ is a new biovar of nodulating bacteria [5].

**Table 1:** Classification and features of *Mesorhizobium ciceri* bv. *biserrulae* strain WSM1271^T^ according to the MIGS recommendations [8,9].

**MIGS ID**	**Property**	**Term**	**Evidence code**
	Current classification	Domain *Bacteria*	TAS [9]
		Phylum *Proteobacteria*	TAS [10]
		Class *Alphaproteobacteria*	TAS [11,12]
		Order *Rhizobiales*	TAS [11,13]
		Family *Phyllobacteriaceae*	TAS [11,14]
		Genus *Mesorhizobium*	TAS [15]
		Species *Mesorhizobium ciceri* bv *biserrulae*	TAS [15]
			
	Gram stain	Negative	TAS [6]
	Cell shape	Rod	TAS [6]
	Motility	Motile	TAS [6]
	Sporulation	Non-sporulating	TAS [16]
	Temperature range	Mesophile	TAS [16]
	Optimum temperature	28°C	TAS [6]
	Salinity	Unknown	NAS
MIGS-22	Oxygen requirement	Aerobic	TAS [16]
	Carbon source	Arabinose, β-gentibiose, glucose, mannitol & melibiose	TAS [6]
	Energy source	Chemoorganotroph	TAS [16]
MIGS-6	Habitat	Soil, root nodule, host	TAS [6]
MIGS-15	Biotic relationship	Free living, Symbiotic	TAS [6]
MIGS-14	Pathogenicity	None	NAS
	Biosafety level	1	TAS [17]
	Isolation	Root nodule	TAS [5,6]
MIGS-4	Geographic location	5 km before Bottida, Sardinia	TAS [2,5]
MIGS-5	Nodule collection date	April 1993	TAS [4]
MIGS-4.1	Longitude	9.012008	NAS
MIGS-4.2	Latitude	40.382709	NAS
MIGS-4.3	Depth	10 cm	NAS
MIGS-4.4	Altitude	295 m	TAS [5]

Evidence codes - TAS: Traceable Author Statement (i.e., a direct report exists in the literature); NAS: Non-traceable Author Statement (i.e., not directly observed for the living, isolated sample, but based on a generally accepted property for the species, or anecdotal evidence). Evidence codes are from the Gene Ontology project [18].

### Symbiotaxonomy

*M. ciceri* bv. *biserrulae* strain WSM1271^T^ has an extremely narrow legume host range for symbiosis only forming highly effective nitrogen-fixing root nodules on *Biserrula pelecinus.* *L.* This strain also nodulates the closely related species *Astragalus membranaceus* but does not nodulate 21 other legume species nodulated by *Mesorhizobium* spp [5]. The high degree of specificity in the symbiotic relationships of this strain is representative of root nodule bacteria isolated from *B. pelecinus* L. growing in undisturbed landscapes in the Mediterranean basin, and is an important example of a highly specific legume host-root nodule bacteria relationship in an annual herbaceous legume used as a forage species in agriculture.

## Genome sequencing and annotation

### Genome project history

The Joint Genome Institute (JGI) operated by US Department of Energy (DOE) sequenced, finished and annotated WSM1271 as part of the Community Sequencing Program (CSP). The genome project is deposited in the Genomes OnLine Database [21]. The finished genome sequence is in GenBank. The CSP selects projects on the basis of environmental and agricultural relevance to issues in global carbon cycling, alternative energy production, and biogeochemical importance. Table 2 summarizes the project information.

**Table 2:** Genome sequencing project information for *Mesorhizobium ciceri* bv. *biserrulae* strain WSM1271^T^

MIGS ID	Property	Term
MIGS-31	Finishing quality	Finished
MIGS-28	Libraries used	Illumina GAii shotgun library, 454 Titanium standard library and paired end 454 libraries
MIGS-29	Sequencing platforms	Illumina and 454 technologies
MIGS-31.2	Sequencing coverage	454 (26.8x) and Illumina (124x)
MIGS-30	Assemblers	Newbler, version 2.3 and Velvet version 0.7.63, PHRAP and CONSED
MIGS-32	Gene calling method	Prodigal, GenePrimp
	Genbank ID	CP002447 CP002448
	Genbank Date of Release	November 10, 2012
	GOLD ID	Gc01578
	NCBI project ID	48991
	Database: IMG	649633066
	Project relevance	Symbiotic nitrogen fixation, agriculture

### Growth conditions and DNA isolation

*M. ciceri* bv. *biserrulae* strain WSM1271^T^ was grown to mid logarithmic phase in TY rich medium [22] on a gyratory shaker at 28 °C. DNA was isolated from 60 mL of cells using a CTAB (Cetyl trimethyl ammonium bromide) bacterial genomic DNA isolation method [23].

### Genome sequencing and assembly

The Joint Genome Institute (JGI) generated the draft genome of *M. ciceri* bv. *biserrulae* WSM1271^T^ using a combination of Illumina [24] and 454 technologies [25]. The sequencing of an Illumina GAii shotgun library generated 23,461,369 reads totaling 844.6 Mb, a 454 Titanium standard library which generated 277,881 reads and a paired end 454 libraries with average insert size of 1.137 +/- 2.842 Kb and 4.378 +/- 1.094 kb which generated 40,653 and 130,843 reads totaling 244.0 Mb of 454 data. All general aspects of library construction and sequencing performed at the JGI can be found at the JGI website [23]. The initial draft assembly contained 32 contigs in 2 scaffolds. The 454 Titanium standard data and the 454 paired end data were assembled together with Newbler, version 2.3. The Newbler consensus sequences were computationally shredded into 2 Kb overlapping fake reads (shreds). Illumina sequencing data was assembled with VELVET, version 0.7.63 [26], and the consensus sequences were computationally shredded into 1.5 Kb overlapping fake reads (shreds). We integrated the 454 Newbler consensus shreds, the Illumina VELVET consensus shreds and the read pairs in the 454 paired end library using parallel phrap, version SPS - 4.24 (High Performance Software, LLC). The software Consed [27-29] was used in the following finishing process. Illumina data was used to correct potential base errors and increase consensus quality using the software Polisher developed at JGI (Alla Lapidus, unpublished). Possible mis-assemblies were corrected using gapResolution (Cliff Han, unpublished), Dupfinisher [30], or sequencing cloned bridging PCR fragments with subcloning. Gaps between contigs were closed by editing in Consed, by PCR and by Bubble PCR (J-F Cheng, unpublished) primer walks. A total of 49 additional reactions were necessary to close gaps and to raise the quality of the finished sequence. The total size of the genome is 6,890,027 bp and the final assembly is based on 112.0 Mb of 454 draft data which provides an average 26.8× coverage of the genome and 832.1 Mb of Illumina draft data which provides an average 124× coverage of the genome.

### Genome annotation

Genes were identified using Prodigal [31] as part of the Oak Ridge National Laboratory genome annotation pipeline, followed by a round of manual curation using the JGI GenePrimp pipeline [32]. The predicted CDSs were translated and used to search the National Center for Biotechnology Information (NCBI) non-redundant database, UniProt, TIGRFam, Pfam, PRIAM, KEGG, COG, and InterPro databases. These data sources were combined to assert a product description for each predicted protein. Non-coding genes and miscellaneous features were predicted using tRNAscan-SE [33], RNAMMer [34], Rfam [35], TMHMM [36], and SignalP [37]. Additional gene prediction analyses and functional annotation were performed within the Integrated Microbial Genomes (IMG-ER) platform [38].

## Genome properties

**Figure 5:**
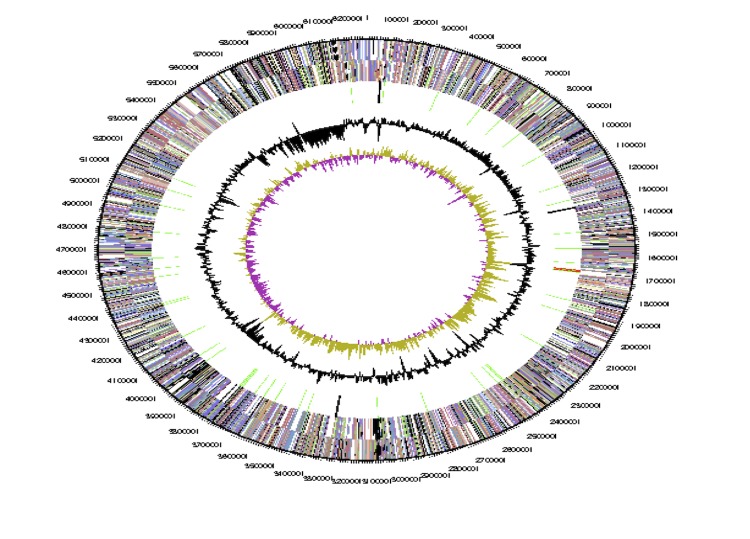
Graphical circular map of the chromosome. From outside to the center: Genes on forward strand (color by COG categories as denoted by the IMG platform), Genes on reverse strand (color by COG categories), RNA genes (tRNAs green, sRNAs red, other RNAs black), GC content, GC skew.

**Figure 6:**
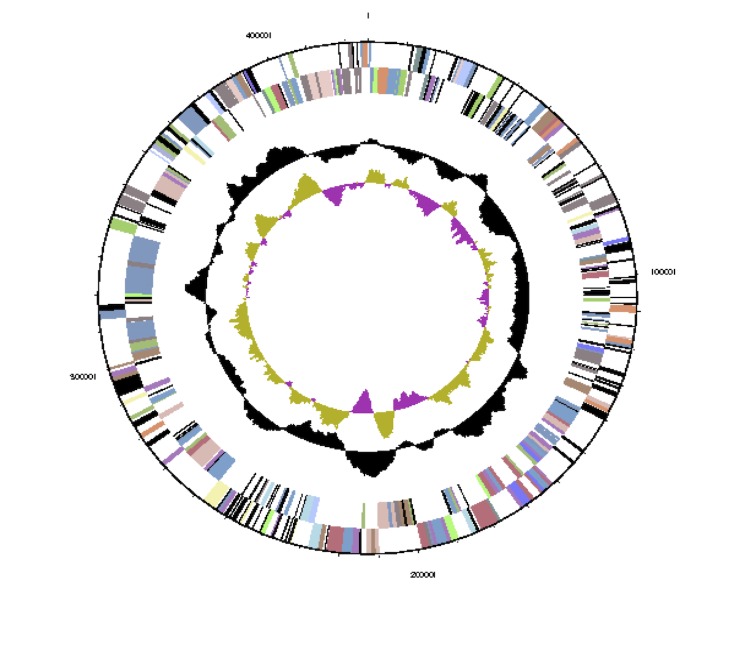
Graphical circular map of the plasmid of *Mesorhizobium ciceri* bv. *biserrulae* WSM1271^T^. From outside to the center. Genes on forward strand (color by COG categories as denoted by the IMG platform), Genes on reverse strand (color by COG categories), RNA genes (tRNAs green, sRNAs red, other RNAs black), GC content, GC skew.

**Figure 7:**
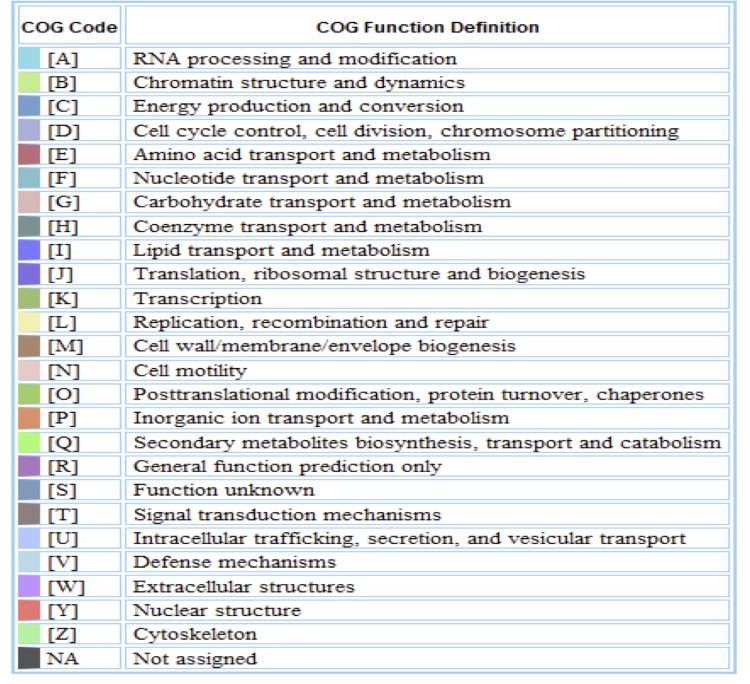
Color code for Figure 5 and 6.

The genome is 6,690,028 bp long with a 62.56% GC content (Table 3) and comprises a single chromosome and a single plasmid. From a total of 6,531 genes, 6,470 were protein encoding and 61 RNA only encoding genes. Within the genome, 206 pseudogenes were also identified. The majority of genes (70.74%) were assigned a putative function while the remaining genes were annotated as hypothetical. The distribution of genes into COGs functional categories is presented in Table 4, and Figures 5,6 and 7.

**Table 3:** Genome Statistics for *Mesorhizobium ciceri* bv. *biserrulae* strain WSM1271^T^.

**Attribute**	**Value**	**% of Total**
Genome size (bp)	6,690,028	100.00
DNA coding region (bp)	5,791,860	86.57
DNA G+C content (bp)	4,185,397	62.56
Number of replicons	2	
Extrachromosomal elements	1	
Total genes	6,531	100.00
RNA genes	61	0.93
Protein-coding genes	6,470	99.07
Genes with function prediction	4,620	70.74
Genes assigned to COGs	5174	79.22
Genes assigned Pfam domains	5398	82.65
Genes with signal peptides	597	9.14
Genes with transmembrane helices	1528	23.40

**Table 4:** Number of protein coding genes of *Mesorhizobium ciceri* bv. *biserrulae* WSM1271^T^ associated with the general COG functional categories.

**Code**	**Value**	**%age**	**COG Category**
J	193	3.35	Translation, ribosomal structure and biogenesis
A	1	0.02	RNA processing and modification
K	492	8.53	Transcription
L	156	2.71	Replication, recombination and repair
B	6	0.10	Chromatin structure and dynamics
D	35	0.61	Cell cycle control, mitosis and meiosis
Y	0	0.00	Nuclear structure
V	63	1.09	Defense mechanisms
T	238	4.13	Signal transduction mechanisms
M	290	5.03	Cell wall/membrane biogenesis
N	62	1.08	Cell motility
Z	0	0.00	Cytoskeleton
W	2	0.03	Extracellular structures
U	124	2.15	Intracellular trafficking and secretion
O	185	3.21	Posttranslational modification, protein turnover, chaperones
C	356	6.17	Energy production conversion
G	535	9.28	Carbohydrate transport and metabolism
E	732	12.70	Amino acid transport metabolism
F	92	1.60	Nucleotide transport and metabolism
H	204	3.54	Coenzyme transport and metabolism
I	235	4.08	Lipid transport and metabolism
P	274	4.75	Inorganic ion transport and metabolism
Q	175	3.04	Secondary metabolite biosynthesis, transport and catabolism
R	731	12.68	General function prediction only
S	585	10.15	Function unknown
-	1,357	20.78	Not in COGS
